# *Plasmodium vivax* Adherence to Placental Glycosaminoglycans

**DOI:** 10.1371/journal.pone.0034509

**Published:** 2012-04-17

**Authors:** Kesinee Chotivanich, Rachanee Udomsangpetch, Rossarin Suwanarusk, Sasithon Pukrittayakamee, Polrat Wilairatana, James G. Beeson, Nicholas P. J. Day, Nicholas J. White

**Affiliations:** 1 Department of Clinical Tropical Medicine, Faculty of Tropical Medicine, Mahidol University, Bangkok, Thailand; 2 Department of Pathobiology, Faculty of Science, Mahidol University, Bangkok, Thailand; 3 Laboratory of Malaria Immunology, Agency for Science, Technology and Research, Singapore, Singapore; 4 Infection and Immunity Division, Walter and Eliza Hall Institute of Medical Research, Parkville, Australia; 5 Centre for Tropical Medicine, Churchill Hospital, Oxford, United Kingdom; University of Copenhagen, Denmark

## Abstract

**Background:**

*Plasmodium vivax* infections seldom kill directly but do cause indirect mortality by reducing birth weight and causing abortion. Cytoadherence and sequestration in the microvasculature are central to the pathogenesis of severe *Plasmodium falciparum* malaria, but the contribution of cytoadherence to pathology in other human malarias is less clear.

**Methodology:**

The adherence properties of *P. vivax* infected red blood cells (*Pv*IRBC) were evaluated under static and flow conditions.

**Principal Findings:**

*P. vivax* isolates from 33 patients were studied. None adhered to immobilized CD36, ICAM-1, or thrombospondin, putative ligands for *P. falciparum* vascular cytoadherence, or umbilical vein endothelial cells, but all adhered to immobilized chondroitin sulphate A (CSA) and hyaluronic acid (HA), the receptors for adhesion of *P. falciparum* in the placenta. *Pv*IRBC also adhered to fresh placental cells (N = 5). Pre-incubation with chondroitinase prevented *Pv*IRBC adherence to CSA, and reduced binding to HA, whereas preincubation with hyaluronidase prevented adherence to HA, but did not reduce binding to CSA significantly. Pre-incubation of *Pv*IRBC with soluble CSA and HA reduced binding to the immobilized receptors and prevented placental binding. *Pv*IRBC adhesion was prevented by pre-incubation with trypsin, inhibited by heparin, and reduced by EGTA. Under laminar flow conditions the mean (SD) shear stress reducing maximum attachment by 50% was 0.06 (0.02) Pa but, having adhered, the *Pv*IRBC could then resist detachment by stresses up to 5 Pa. At 37°C adherence began approximately 16 hours after red cell invasion with maximal adherence at 30 hours. At 39°C adherence began earlier and peaked at 24 hours.

**Significance:**

Adherence of *P. vivax*-infected erythrocytes to glycosaminoglycans may contribute to the pathogenesis of vivax malaria and lead to intrauterine growth retardation.

## Introduction

Vital organ dysfunction and death from *P falciparum* malaria results from microvascular obstruction caused by the sequestration of red cells containing mature forms of the parasite. *P.falciparum* infections in pregnancy are associated with a consistent reduction in birth weight, particularly in primigravidae. This is a major risk factor for neonatal death. Intrauterine growth retardation is associated with the accumulation of *P.falciparum* infected red cells in the placenta. This accumulation results from the adherence of a specific and relatively conserved red cell surface expressed domain of the antigenically variant membrane protein (PfEMP1) to the placental glycosaminoglycan chondroitin sulphate A, and to secondary receptors such as hyaluronic acid and immunoglobulins [1]–[5]. *Plasmodium vivax* is generally regarded as a more benign parasite than *P.falciparum*, and is associated with a lower mortality, although severe forms may occur occasionally [6]. *P.vivax* accounts for approximately half of all malaria outside Africa [7]. Like *P.falciparum, P.vivax* has also exerted a considerable selective pressure on human evolution although pathological processes are less well understood. *Plasmodium vivax* causes rosetting (adherence to uninfected erythrocytes) [8] but until recently it has not been considered to cytoadhere [9]. *P. vivax* infections in pregnancy also cause abortions [10] and reduce birthweight which increases the risk of neonatal death, although the mechanism underlying early fetal loss or the intrauterine growth retardation is unclear [11]. We have investigated the adherence of *P. vivax* (N = 33) to the placental glycosaminoglycans chondroitin sulphate A (CSA) and hyaluronic acid (HA), the putative receptors for *P. falciparum* placental adherence [10], [11].

## Materials and Methods

### Ethics statement

This study was a part of clinical studies which have been approved by the Ethics Committee, Faculty of Tropical Medicine, Mahidol University. All participants gave fully informed consent to providing a 5 mL blood sample. Written informed consent was provided by study participants.

### Parasites

Synchronous fresh isolates (>80% ring stage) of *Plasmodium vivax* were obtained from non-pregnant adult patients with acute vivax malaria admitted to the Hospital for Tropical diseases, Bangkok and entered into clinical studies. Malaria parasite species were confirmed by PCR [12]. All blood samples were recorded using a code identifier, and the subsequent experiments were conducted and the results were interpreted blinded to the patient data. Blood samples were taken into heparinized tubes and cultured as described previously [13]. Briefly, blood samples were centrifuged at 500 g at 4°C then plasma was discarded. White blood cells were removed by a CF-11 column or Plasmodipur® filter. After 24 hours of cultivation, trophozoite-infected red cells were used for further experiments. The parasite density of clinical isolates with less than 0.5% parasitaemia was augmented by concentration using a 66% Percoll® gradient [14] or a magnetic separation column [15]. The synchronous trophozoite-IRBCs were enriched to 80–90% parasitaemia and the concentrate then adjusted to 1% parasitaemia at 1% haematocrit in PV-MCM media for the static adherence assay [16], [17], and to 2% Haematocrit in 1% albumax for the laminar sheer flow adherence assay. Highly synchronized ring stage parasites (>0.5% parasitaemia) were used for assessing the relationship between adherence and stage of parasite development. The *P. falciparum* A4 clone, selected for adhesion to CSA (kindly provided by Dr David Roberts) was used as the control. Rosette formation (the adherence of two or more uninfected red cells to the infected cells) was counted for 100 IRBCs as described previously [18].

### Static adherence assay

Adherence of parasitized erythrocytes to umbilical vein endothelial cells was assessed as described previously [19]. The reagents evaluated as potential receptors in the adherence assay were: purified CD36 and ICAM-1 (kindly provided by Arnab Pain), Thrombospondin (TSP; provided by Rachanee Udomsangpetch), CSA (from bovine trachea Sigma@ cat no C8529, or CSA covalently linked to phosphatidylethanolamine, kindly provided by Stephen Rogerson), CSC (chondroitin sulphate C), de-6-O-sulphated CSA [20], dextran sulphate with molecular weight of 500 kD (Sigma), and HA (from bovine vitreous humor, Sigma ^@^ cat no H7630). Assessment of adherence to these receptors was performed as described previously [20]. Briefly, receptors (at 100 ug/mL) were coated on plastic Petri dishes for 24 hours at 4°C, and then blocked by 1% bovine serum albumin in phosphate buffer saline (PBS) before being used. The IRBC suspension was incubated with the immobilized receptor spots for 30 minutes, at 37°C. After that, unbound red cells were removed by gently washing with RPMI-HEPES. The adherent cells were fixed with glutaraldehyde, and stained with Giemsa. The number of IRBCs bound was counted per 100 high power fields or 1 mm^2^.

### Factors reducing *P. vivax*-infected red cell adherence

Soluble CSA (50 ug/mL) and HA (50 ug/mL) were incubated with the IRBC suspensions for 15 minutes at 37°C and the red cells then resuspended before testing adherence**.** In order to characterize further the adherence properties of *Pv*IRBC, the effects of heparin (Leo^®^ 1–1000 units/mL) and EGTA (0.01–1 mM) on adherence were assessed by coincubation for 2 hours at 37°C before the adherence assays were performed. In separate experiments, parasite cultures were coincubated with trypsin (1 to 1000 ug/mL; Sigma) for 15 minutes at 37°C, then washed and resuspended in PV-MCM culture medium containing 10% human serum and tested in the adherence assay as described above. *P.vivax* adherence to HA and CSA was evaluated further by incubating the Petri dishes with chondroitinase ABC (5 units/mL) and testicular hyarulonidase (10 unit/mL) or without enzyme (control) at 37°C for 45 minutes. The number of bound IRBCs was counted per 100 high power fields or 1 mm^2^. The median and ranges of adherence cell numbers were calculated.

### Stage of P. vivax development and adherence to CSA and HA

The relationship between *P. vivax* IRBC adherence and stage of parasite development was investigated in ten isolates during the 48 hour asexual blood stage cycle *in vitro*. Parasite ring stages (6 hour old) were cultured as described previously [13], in 5% CO_2_ at 37 °C and 39°C in parallel. Thin blood films were prepared every 6 hours up to 42 hours of parasite development and stained with Field's stain. In the static adherence assay the number of CSA and HA adherent IRBCs per 1000 red cells was counted every six hours and parasite developmental stage evaluated in 100 infected cells using staging criteria described previously [13].

### *P. vivax* IRBC adherence to fresh placental sections

The Stamper-Woodruff adherence assay [21] was performed with some modification for the assessment of placental adhesion. Freshly frozen normal placenta sections (10 μm thick) were placed on glass slides and fixed with 3% glutaraldehyde in PBS for 30 minutes, then washed with RPMI-1640, kept wet and then placed on a tray. The concentrated *P.vivax* infected red cell (*P.v* IRBC) suspension at 80% parasitaemia and 1% haematocrit was overlaid on the slides and incubated for 30 minutes at 37°C in 5%CO_2_. After incubation, non-adherent cells were removed by rinsing with RMPI-1640. The remaining adherent cells were fixed with 3% glutaraldehyde in PBS and stained with Giemsa. Placenta-adherent *P.v* IRBCs binding in the intervillous space and along the syncytiotrophoblast layer were counted under light microscopy from 50 high power fields. Uninfected red cell suspensions (1% haematocrit) overlaid on the placenta sections served as controls in each assay. The specific adhesion of the infected red cells on placenta was further investigated by incubating IRBC suspensions with soluble CSA (50 ug/mL) and HA (50 ug/mL) for 15 minutes at 37°C before testing adherence.

### *Pv* IRBC adherence under laminar flow conditions

The strength of *P.vivax* infected red cell adhesion was assessed under laminar flow conditions. An infusion/withdrawal pump (Harvard II apparatus®,) infused the cell suspension through microslides (placed on the stage of the inverted microscope) at defined flow rates and hence, defined wall shear stresses (calculated from the dimensions of the microslides and the flow rate, the temperature and the viscosity of the suspension.) as described previously [22]. The microslides were coated with 5 mg/mL poly-L-lysine for 30 minutes, then coated overnight with CSA [22], [23]. The coated slides were blocked with 1% bovine serum albumin in PBS before performing the adherence assays. PBS was used as the control. Red cell suspensions (1% albumax® in RPMI-1640) containing 2×10^8^ red cells/mL and *P.vivax* trophozoites (∼30 hours old) at 0.5% parasitaemia flowed through the microslides at a shear stress of 0.05 Pa, for 5 minutes. Non-adherent cells were removed by perfusion with 1% albumax in RPMI-1640 for 5 minutes. After removing the non-adherent cells, the adherent cells were counted per 120 mm^2^ surface area. Six *P.vivax* isolates were evaluated. CSA-adherent *P. falciparum* parasites derived from clone A4, were used as positive controls. The force of adhesion was then assessed under increasing shear stresses (0.01 to 8 Pa.). The already adherent cells (10 infected cells per isolate) were exposed to step-wise increases in shear stress until the cells were detached.

### Statistical methods

Comparisons were performed using the Mann Whitney U test, proportions using the Chi-squared test and the correlation between the levels of adherence to different receptors was assessed using the Spearman Rank correlation coefficient. These were calculated using SPSS (version 11.5).

## Results

In total 33 fresh *P. vivax* isolates were obtained from adults with acute vivax malaria. Admission parasitaemias ranged from 0.3–2.8%. *P.falciparum* coinfection was excluded by both microscopy and PCR genotyping in each case. The mean (SD) parasitaemia was 0.7 (0.3) %. Of 33 isolates 30 (93%) formed rosettes. The mean (SD) number of infected red cells (IRBCs) forming rosettes was 21 (17) per 100 infected red cells.

**Figure 1:**
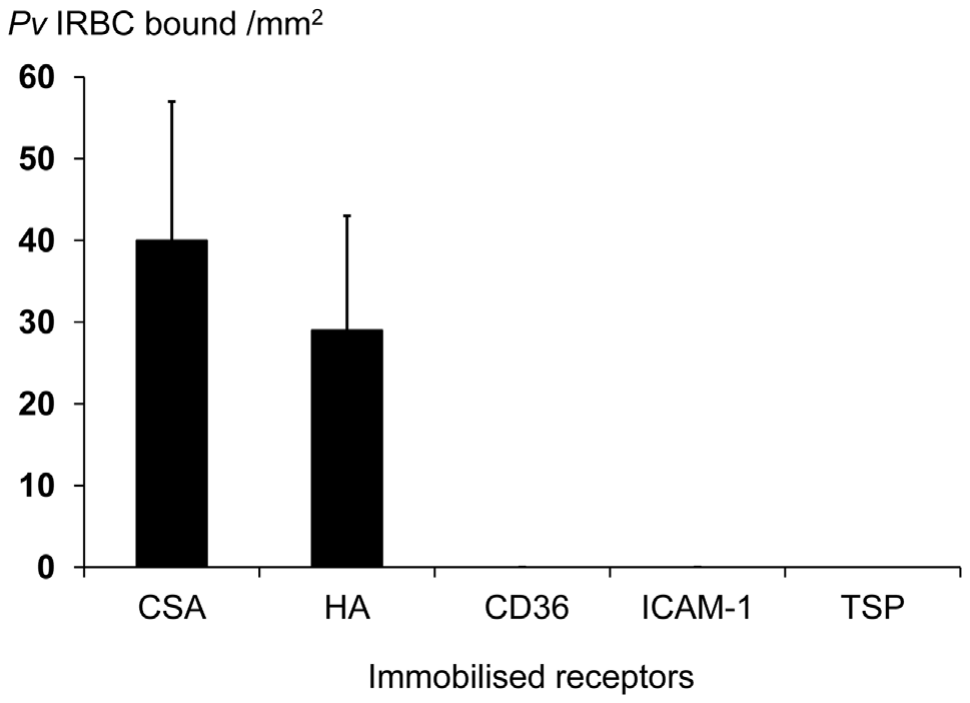
Numbers of *P.vivax* infected erythrocytes bound to immobilised potential cytoadherence receptors. HA; hyaluronic acid, CSA: chondroitin sulphate A, TSP: thrombospondin. Numbers are mean values and error bars are standard deviations.

### Binding to putative receptors

**Figure 2:**
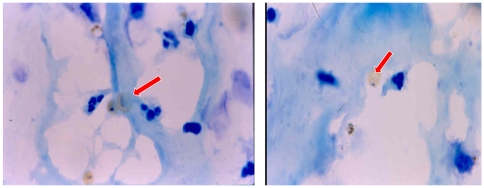
*P.vivax* infected red blood cells (arrows) adherent to the surface of fresh placenta.

None of the *P.v*IRBCs adhered to immobilized CD36, ICAM-1, or thrombospondin, the putative vascular receptors for *P.falciparum,* whereas all 33 *P.vivax* isolates adhered to immobilized CSA and HA. The mean (SD) level of adhesion to CSA was 40 (25) and to HA was 33 (21) *P.vivax* infected red blood cells (*P.v* IRBCs) bound /mm^2^ (Fig. 1). The levels of adherence to CSA and HA were correlated strongly (r_s_ = 0.95; p<0.001). Bland –Altman analysis gave a mean (95% confidence interval) difference of 7.6 (4.65 to 10.54) *P.v* IRBCs bound /mm^2^ from the average adhesion to the two receptors. None of the parasite isolates bound to human umbilical vein endothelial cells (data not shown), chondroitin sulphate-C, or dextran 500. There was no correlation between the number of *P.v* IRBC rosettes formed and adherence to CSA and HA. Adherence of *P. vivax* IRBCs to fresh placenta sections was assessed in five isolates, all of which showed attachment (Fig. 2). The mean (SD) number of bound *P.v* IRBCs per mm^2^ was 52 (19).

### Specificity of *Pv*IRBC adherence

**Figure 3:**
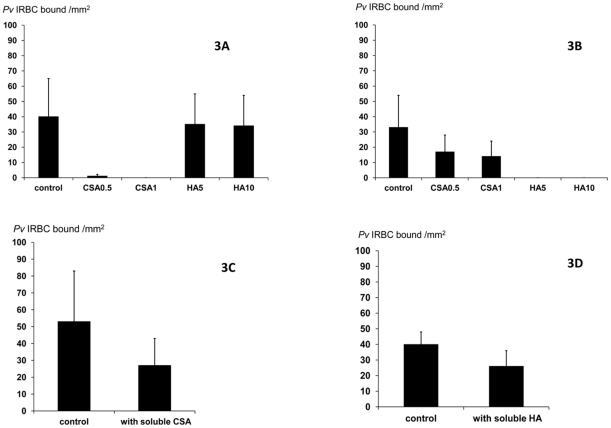
Specific adhesion of *P.vivax* infected red blood cells (*P.v* IRBC) to immobilised potential receptors and the effects of enzyme pretreatment and preincubation with soluble receptors. (**A**) Chondroitin sulphate A (CSA) was pretreated with chondroitinase ABC (0.5, 1 unit/mL). (**B**) Hyaluronic acid (HA) was pretreated with hyaluronidase (5, 10 units /mL). (**C**) *P.v* IRBCs were pre-incubated with soluble CSA (50 ug/mL). (**D**) *P.v* IRBCs were pre-incubated with soluble HA (50 ug/mL). Data are presented as medians with 95% confidence intervals.

In order to determine the specificity of adherence both CSA and HA were pretreated with chondroitinase ABC (0.5 and 1 unit/mL) or hyaluronidase (5 and 10 units/mL) (n = 21). Chondroitinase ABC at 0.5 unit/mL almost abolished *P.v* IRBC adherence to CSA (median 98%; range 91–100%; p<0.01) and reduced binding to HA (median 50%; range 15–77%; p<0.01). Hyaluronidase completely inhibited *P.v* IRBC binding to HA, not to CSA (p<0.01; Fig. 3A, B). Adherence to CSA (n = 7) was also inhibited by pre-incubation with soluble CSA (50 ug/mL); median 75% (range 52–95%); p<0.01, and adherence to HA (n = 7) was inhibited by soluble HA (10 ug/mL); median 71% (range 61–90%) (p<0.01; Fig. 3C, D).

### Effects of trypsin, heparin and EGTA

**Figure 4:**
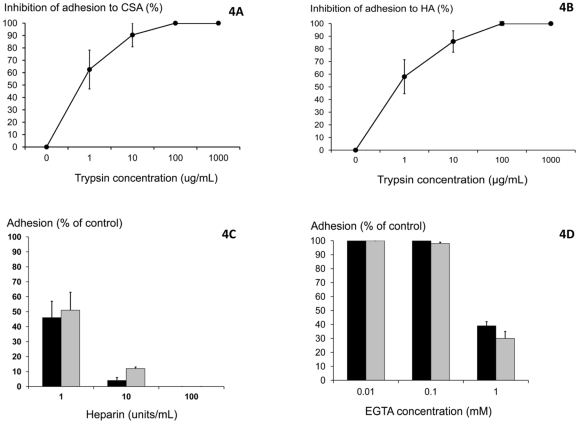
Effects of trypsin pre-incubation, heparin and EGTA on the adhesion of *P.vivax* infected red blood cells to immobilised CSA and HA. (A) Effects of pre-incubation with trypsin on adhesion to CSA. (B) Effects of pre-incubation with trypsin on adhesion to HA. (C) Effects of heparin on binding to immobilized CSA and HA. Black bars: CSA, Grey bars: HA. (D) Effects of EGTA on binding to immobilized CSA and HA. Black bars: CSA, Grey bars: HA. Data are presented as medians with 95% confidence intervals.

Proteolysis of *P.v* IRBC surface proteins by high concentrations of trypsin (>10 ug/mL) completely inhibited adherence. Lower concentrations (1–10 ug/mL) decreased *P.v* IRBC binding to CSA (n = 32) and HA (n = 20) by more than 50% (ranges 30–99% and 38–90%, respectively) (p<0.01; Fig. 4A, B). Heparin inhibited *P.v* IRBC adherence to CSA and HA; 50% inhibition of adherence to CSA and HA was obtained with 1 unit/mL of heparin (n = 32; range of inhibition 41 to 65%) and >10 unit/mL completely inhibited adherence (p<0.01; Fig. 4C). *P. vivax* isolates did not bind to heparin coated plates. High concentrations (1 mM) of the calcium chelator EGTA inhibited *P.v* IRBC adherence to CSA; median 60% range 57–63% (n = 32), and to HA; median 70% range 65–75% (n = 20) (p<0.01; Fig. 4D), but lower concentrations (0.01–0.1 nM) had no significant effects.

### Effects of parasite incubation temperature on adhesion properties

**Figure 5:**
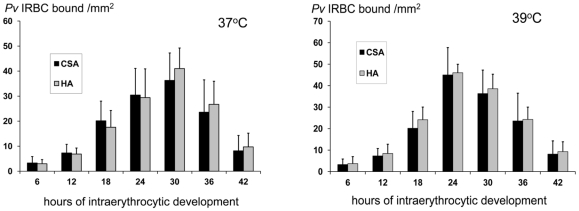
The effects of temperature on the development of *Plasmodium vivax* adherence properties. (**A**) Asexual development in synchronous *in-vitro* culture at 37°C, the peak of adhesion appears at approximately 30 hours of intraerythrocytic development. (**B**) At 39°C, peak adhesion appears at approximately 24 hours of intraerythrocytic development.

At an incubation temperature of 37°C adherence to CSA and HA began when the *P.vivax* parasites reached the large ring stage of development (approximately 12–16 hours after invasion). Adherence reached mean (SD) maximum values at 30 hours (n = 10); CSA 36 (10) and HA 41 (8) *P.v* IRBCs bound per mm^2^ respectively. At 39°C adherence began earlier and reached peak values at 24 hours; CSA 45 (2) and HA 46 (3) per mm^2^ respectively (Fig. 5A, B).

### *P.v* IRBC adhesion under laminar flow conditions

**Figure 6:**
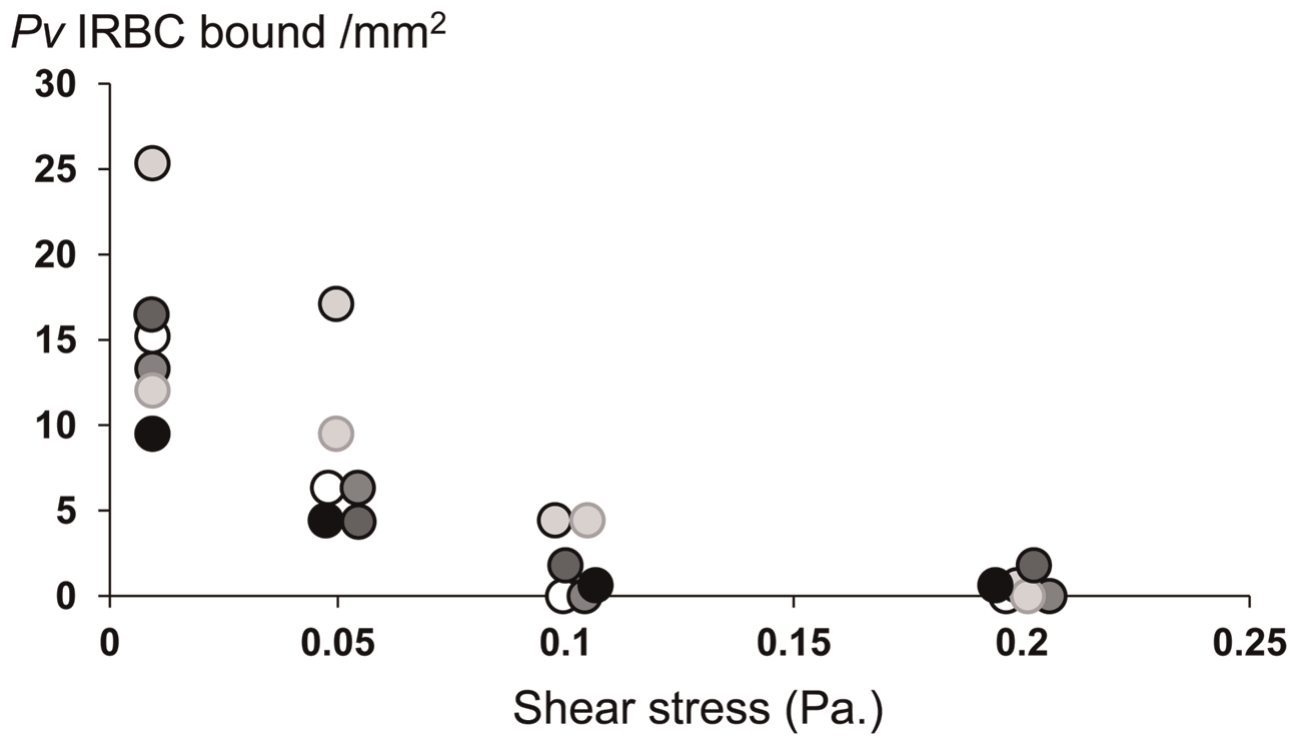
Relationship of shear stresses to the adherence of *P.v* IRBCs to CSA under laminar flow conditions (6 isolates). Data are presented as means and standard deviations.

In the *in vitro* laminar flow assay *P.v* IRBC adherence to CSA occurred only at low shear stresses (6 parasite isolates; 40 *P.v* IRBC each assessed). Maximum values were obtained at 0.01 Pa (Fig. 6). There was less adhesion at 0.1 Pa and none when shear stresses increased to 0.2 Pa. The mean (SD) shear stress reducing maximum attachment by 50% was 0.06 (0.02) Pa. However once they had adhered the *P.v* IRBCs were more resistant to mechanical detachment; most (34 out of 40) were detached after exposed to 1 Pa shear stresses (for 5 minutes), but 6 out of 40 *P.v* IRBCs resisted detachment by shear stresses of up to 5 Pa, for 5 minutes. Pre-incubation of *Pv* IRBCs with soluble CSA before perfusion (n = 3) totally inhibited parasite binding (p<0.01).

## Discussion

*Plasmodium vivax* is generally considered to produce debilitating but non-fatal relapsing infections. Recent case series suggest this relatively benign reputation may need some revision [6]. Low birth weight, malnutrition, developmental retardation, severe anaemia, acute pulmonary oedema and encephalopathy are all well documented with vivax malaria [6], [24]–[26]. *P.vivax* has not previously been considered to sequester in the microcirculation through cytoadherence of infected erythrocytes, although recent physiological studies of pulmonary gas transfer in vivax malaria might be explained by pulmonary sequestration [27]. Consequently severe manifestations have been generally ascribed to other pathological processes, such as high systemic concentrations of pro-inflammatory cytokines. This study conducted in Thailand confirms that *Plasmodium vivax* infected erythrocytes do not cytoadhere to the principal vascular endothelial ligands responsible for *P.falciparum* sequestration in the microvasculature, or to umbilical vein endothelial cells, but they do cytoadhere to both CSA and HA, the placental syncytiotrophoblast glycosaminoglycans responsible for *P.falciparum* sequestration in the placenta [1]–[5], [28]–[30]. Similar findings have recently been reported from South America [9]. *P.v* IRBC also adhered to fresh placental cells, although binding was not intense. Malaria reduces birthweight and this reduces infant survival [11], [31]. In *P.falciparum* infections this is associated with placental sequestration [1], [2]. The data presented here suggest that the adverse effects of vivax malaria on intrauterine development may have a similar pathogenesis, and raise the possibility that pathology in other organs (such as lung capillaries and venules in patients who develop pulmonary oedema) might also result from vascular endothelial adhesion to chondroitin sulphate (CSA).

All isolates bound both CSA and HA to some extent although the degree of adhesion to the two receptors was different with some isolates. The specificity of adherence to these glycosaminoglycan receptors is supported by the inhibition by pretreatment with hyaluronidase and chondroitinase and competitive blocking by the soluble purified glycosaminoglycans under static and flow conditions [28]–[30]. Soluble CSA blocked binding to placental sections supporting a primary role for this molecule in placental cytoadherence. Commercial preparations of HA have been contaminated by CSA so the precise nature of the interaction with these two glycosaminoglycans remains to be elucidated. *P.v*IRBC adhesion to CSA and HA was inhibited by heparin, but was relatively resistant to cation depletion (EGTA), suggesting charge might contribute to non-specific inhibitory effects. *P.falciparum* cytoadherence is prevented by trypsin. *Pv*IRBC adherence to CSA and HA was also largely abolished by trypsin indicating a red cell surface protein –receptor interaction [1]–[3], [6], but the identity of this protein remains to be identified. The different sensitivities to trypsin among the different isolates may indicate different levels of adhesive surface protein expression, different adhesins, or multiple domains of the same adhesive molecules, as observed for *P. falciparum* binding to both CD36 and ICAM-1 [32].

*P.v* IRBC cytoadherence had other properties which are similar to that of *P.falciparum.* The IRBCs began to cytoadhere at the end of the first third of the asexual cycle, at the large ring to trophozoite stage, and this was accelerated by incubation at a “febrile” temperature (39°C) [33]. Cytoadherence did not occur at high shear stresses, but once the IRBCs had adhered to CSA or HA they were relatively resistant to increases in shear stresses up to 5 Pa.

*P.vivax* reduces birthweight but the causative mechanisms have been unclear. *P.vivax* forms rosettes more than *P.falciparum*, and this might also contribute to pathological processes in the placenta. There is very limited information on placental pathology in acute vivax malaria so the extent of sequestration in-vivo is not known [34], [35]. Adhesion of *Plasmodium vivax* infected red cells occurred at the low shear stresses (0.01–0.06 Pa) similar to those encountered in the intervillous spaces of the placenta. Higher stresses prevail in the systemic circulation. Given the physical characteristics of the placental circulation with its large vascular spaces, mechanical obstruction seems unlikely to account for placental dysfunction. Inflammatory processes with secondary interference with nutrient transfer may be more likely. Whichever the mechanism the evidence from *P. falciparum* infections that glycosaminoglycans are an important contributor to placental cytoadherence, and that antibodies which block this adherence are associated with protection against the adverse effect of birth weight, strongly suggests a pathological role [36]. The Duffy binding ligand of *P.falciparum* PfEMP1 that mediates cytoadherence to CSA is being developed as a possible vaccine against pregnancy malaria. A similar strategy might prevent the adverse effects of vivax malaria in pregnancy.
